# Personalized sirolimus regimen for vascular malformations: a retrospective analysis of VASE cohort

**DOI:** 10.1186/s13023-025-04151-y

**Published:** 2025-12-10

**Authors:** Emmanuel Seront, An Van Damme, Julien Coulie, Miikka Vikkula, Laurence M. Boon

**Affiliations:** 1 Department of Medical Oncology, Institut Roi Albert II, Saint-Luc University Hospital, UCLouvain, Brussels, Belgium; 2 Department of Pediatic Hemato-oncology, Institut Roi Albert II, Saint-Luc University, UCLouvain, Brussels, Belgium; 3 Division of Plastic Surgery, Saint-Luc University Hospital, UCLouvain, Brussels, Belgium; 4 Human Molecular Genetics, de Duve Institute, UCLouvain, Brussels, Belgium; 5 WELBIO department, WEL Research Institute, Avenue Pasteur, 6, Wavre, 1300 Belgium

**Keywords:** Slow-flow vascular malformation, Sirolimus, Intermittent, On-demand, Personalized regimen, Adverse events

## Abstract

**Background:**

The mTOR inhibitor Sirolimus was shown to improve symptoms in patients with slow-flow vascular malformations, but long-term continuous use is limited by cumulative toxicity. A personalized approach with intermittent regimens may offer similar efficacy with fewer adverse effects (AE). This retrospective analysis evaluated the effectiveness and safety of individualized sirolimus strategies in patients who experienced symptom recurrence after completing the 2-year course in the VASE phase III trial. All patients initially resumed continuous sirolimus for 3 months, then transitioned to one personalized regimen based on their pain profiles: intermittent sirolimus 5 days-ON/2 days-OFF (Group A), hybrid intermittent plus on-demand (Group B), or fully on-demand administration triggered by pain or known stressors (Group C).

**Results:**

Thirty adults were included (Group A: *n* = 13; Group B: *n* = 6; Group C: *n* = 11). Across all groups, intermittent, hybrid or on-demand sirolimus maintained pain control comparable to continuous administration, significantly reducing pain intensity, crisis frequency, and crisis duration from baseline. AEs decreased from 73–85% during continuous therapy to 9–33% with intermittent/on-demand regimens, with no reported grade ≥3 event.

**Conclusion:**

Personalized intermittent sirolimus regimens may effectively control symptoms and substantially reduce toxicity in patients with vascular malformations. This strategy supports individualized, long-term therapy and merits prospective validation.

**Trial registration.:**

NCT02638389 and EudraCT 2015-001703-32

## Background

Vascular malformations result from developmental defects in vascular formation during embryogenesis. These anomalies are classified based on blood flow characteristics and the type of vessels involved. Slow-flow vascular malformations include capillary (CM), venous (VM), and lymphatic malformations (LM), while fast-flow malformations involve arteriovenous malformations. These congenital conditions persist throughout life, growing proportionally with the patient and causing a range of clinical issues, including chronic or exacerbated pain, functional impairments, oozing, bleeding, and aesthetic deformities [1].

Advancements in the understanding of slow-flow vascular malformations have underscored the crucial role of the phosphoinositide 3-kinase (PI3K)-Protein kinase B (AKT)-mammalian target of rapamycin (mTOR) signaling pathway in their pathogenesis and progression [2–7]. Consequently, mTOR inhibitors, such as sirolimus, have emerged as key pharmacological agents for treatment. Clinical trials have demonstrated a clear benefit of sirolimus, significantly improving symptoms and quality of life in both adults and children [6, 8, 9]. The European multicentric phase III trial VASE enrolled 250 patients with slow-flow vascular malformations to receive sirolimus at a dose of 2 mg per day in adults and 0.8 mg/m^2^ twice daily in children, for a period of two years. Preliminary results, analyzing 132 patients who had been on sirolimus for at least 12 months or who had stopped treatment prematurely, showed that sirolimus alleviated symptoms in 85% of patients. Notably, some patients experienced a rapid and significant reduction in symptoms, such as pain and bleeding, within just a few days of starting sirolimus [9]. Following protocol-mandated sirolimus discontinuation after two years, 36% of patients required reintroduction of the drug due to symptom recurrence, reminding that sirolimus is usually not curative and that a number of patients require long-term treatment. Additionally, although adverse events were generally mild, treatment interruptions occurred in up to 34% of patients, with 18% experiencing grade 3–4 toxicities [8].

Balancing the benefits of sirolimus with the need for chronic treatment and associated toxicity highlights the need for personalized therapeutic approaches. This study explores the feasibility of an intermittent or personalized sirolimus administration strategy tailored to individual patient needs.

## Methods

### Study objective

The aim of this study was to assess the feasibility and effectiveness of personalized (intermittent and/or on-demand) sirolimus dosing strategies in adults with slow-flow vascular malformations. The primary endpoint was change in pain outcomes, including continuous pain intensity, pain crisis frequency, and pain crisis duration. Only adult patients enrolled.

### Study design and patient selection

This retrospective analysis included adult patients with slow-flow vascular malformations who had previously completed a 2-year course of continuous sirolimus (2 mg/day) in the VASE phase III clinical trial (ClinicalTrials.gov NCT02638389; EudraCT 2015-001703-32).

Per the VASE protocol, sirolimus could be resumed during follow-up in case of symptom recurrence, primarily pain. All patients in this retrospective analysis experienced symptom recurrence after discontinuation and restarted sirolimus at 2 mg/day for a 3-month reintroduction phase, with dose adjustments after one month to maintain trough levels of 10–15 ng/mL.

Group allocation was determined during this reintroduction phase, based on symptom profiles assessed during the prior washout period—specifically pain intensity, frequency, and the presence of continuous or episodic pain with identifiable triggers.

Following stabilization, patients were transitioned to their personalized dosing regimens. No sirolimus levels were measured during the adapted phases, which were assumed to result in low, time-dependent exposure. The data collection started in January 2020 and ended in December 2024.

### Intermittent and on-demand sirolimus regimens

- Group A (intermittent, five days-ON/two days-OFF): patients with persistent daily pain (VAS > 3) and more than six pain crises/month (VAS > 6) began with 2 mg/day sirolimus, five days per week with two non-consecutive off-days (e.g., Thursday and Saturday; Group A1). After 6 months, those with stable symptoms were offered a reduced dose of 1 mg/day using the same schedule (Group A2). Patients with worsening symptoms reverted to their prior regimen.
- Group B (hybrid intermittent + on-demand): patients with VAS > 3 and ≤ 6 crises/month were treated with 1 mg/day (five days-ON/two days-OFF) and instructed to take an additional 2 mg sirolimus either on the day before and the day of the anticipated trigger, or at the onset of a pain crisis and again the following day. Triggers included physical activity, alcohol consumption, or emotional stress.
- Group C (fully on-demand): patients with VAS ≤3 and ≤ 6 crises/month received no baseline sirolimus but were instead stratified into two subgroups based on the nature of their pain triggers. The Group C1 included patients with predictable crises (e.g., physical exertion, alcohol) who took 2 mg sirolimus the day before and the day of the trigger. The group C2 included patients with unpredictable crises who took 2 mg at pain onset and again the following day. Those with inadequate symptom control reverted to continuous sirolimus.

### Outcome measures

Pain scores were assessed in all groups at multiple time points: prior to sirolimus reintroduction, three months after reintroduction of the continuous sirolimus therapy, at 6 month and 12 months following the initiation of the intermittent regimen, and at 12 month following the initiation of the on-demand and the hybrid strategy.

For these groups, the VAS scores were assessed for daily chronic pain, as well as the frequency (per month) and duration (in days) of pain crises, defined as VAS scores > 6.

All patients included in the study had a minimum follow-up duration of 12 months on their respective adapted treatment regimen. Each patient served as their own control.

Adverse events (AEs) were recorded throughout the study and graded according to the Common Terminology Criteria for Adverse Events (CTCAE) v5.0.

### Statistical analysis

Descriptive statistics summarized baseline characteristics, treatment regimens, and adverse event profiles. Comparative analyses included paired t-tests to compare pain scores across time points.

## Results

**Fig. 1:**
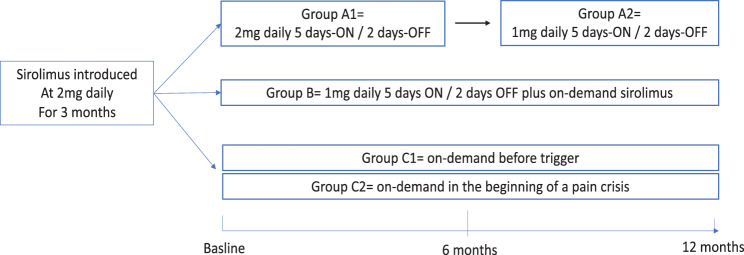
Personalized sirolimus reintroduction strategy. Patients were allocated to intermittent 5 days ON / 2 days off (2 mg in group A1 and 1 mg in group A2) hybrid associating 5 days ON / 2 days off and on-demand (group B) on-demand regimens. (group C1: on-demand before trigger and group C2: on-demand in the beginning of pain crisis)

Thirty adult patients (median age: 32 years; range: 19–78) were included in this analysis. They all experienced symptom improvement during an initial 3-month sirolimus reintroduction phase and were then transitioned to personalized intermittent regimens. Trough sirolimus levels during reintroduction were < 5 ng/mL in 5 patients, 6–10 ng/mL in 7 patients, and 11–15 ng/mL in 18 patients. Thirteen patients were included in Group A, six in Group B and 11 in Group C. The median follow-up was 18 months (range: 15–36 months) after initiating the intermittent regimen. The trial design is summarized in Fig. 1, and patient characteristics are detailed in Table1.

**Table 1:** Demographic characteristics

	Group A *N* = 13	Group B *N* = 6	Group C1 *N* = 5	Group C2 *N* = 6
Median Age	43	32	25	31
(range)	23–78	22–47	19–30	25–68
Sex F:M	9:4	5:1	1:4	3:3
Type of malformation				
- VM	−11	−2	−5	−3
- LM	− 1	− 3	− 0	− 2
- CVM	− 1	− 0	− 0	− 0
- CM	− 0	− 0	− 0	− 1
- KTS	− 0	− 1	− 0	− 0
Mutated gene				
- TIE2	−2	−0	−1	−0
- PIK3CA	− 2	− 3	− 1	− 0
- PTEN	− 1	− 0	− 1	− 0
- GNAQ	− 0	− 0	− 0	− 1
- Unknown	− 8	− 3	− 2	− 5
Anatomical involvement				
- Limb	−10	−6	−3	−3
- Head and neck	− 2	− 0	− 1	− 3
- Thoracic area	− 1	− 0	− 1	− 0
Interval between sirolimus arrest (per VASE protocol) and sirolimus reintroduction				
- < 6 months	−5	−2	−0	−0
− 6–12 months	− 3	− 1	− 0	− 1
- > 12 months	− 5	− 3	− 5	− 5
Serum level of sirolimus during the initial phase	0–5 = 2	0–5 = 1	0–5 = 1	0–5 = 1
	6–10 = 4	6–10 = 1	6–10 = 1	6–10 = 1
	11–15 = 7	11–15 = 4	11–15 = 3	11–15 = 4
Non-responding patients on intermittent regimen				
- n (%)	−3 (23)	−1 (17)	None	−1 (17)
- type of vascular malformation	− 3 VM	- VM		- LM
- localization	- Cervicofacial, lower limb and thoracic	-Lower limb		- Lower limb
- localized/extensive	- all extensive	- extensive		- all extensive
- superficial/deep	- All deep	- deep		- deep
- type of genomic anomaly	- unknown	- unknown		- unknown
- interval between sirolimus arrest and reintroduction (months)	- < 6	- > 12		- > 12

### Intermittent administration of sirolimus: five days-ON/two days-OFF in group A

**Fig. 2:**
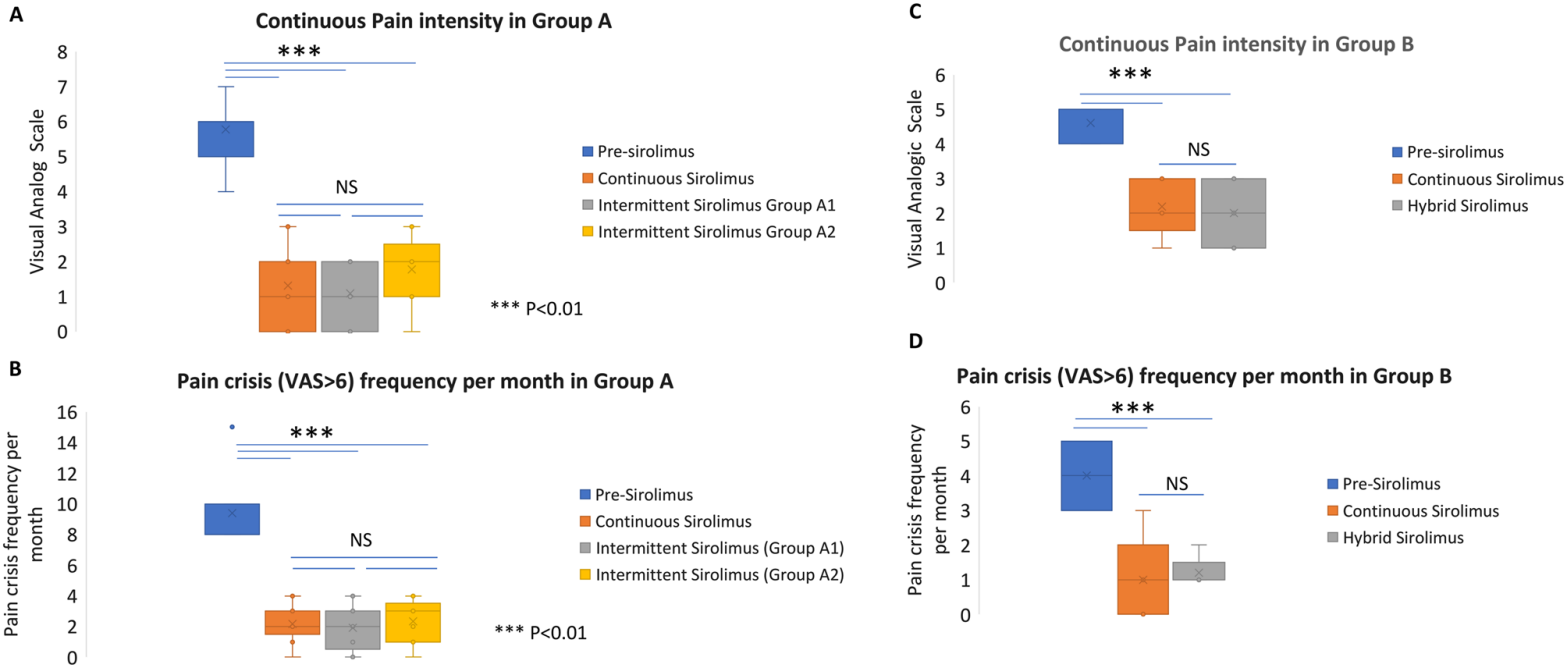
Evolution of symptoms on personalized sirolimus in group A and B Pain intensity (**A**) and crisis frequency (**B**) in Group A before sirolimus, three months after the continuous treatment period, and under stepwise intermittent dosing (Group A1: 2 mg 5d/7; Group A2: 1 mg 5d/7). Symptom control was maintained with reduced dosing. *** *P*<0.001, NS non statistically significant Pain intensity (**C**) and crisis frequency (**D**) in Group B before sirolimus, three months after the continuous treatment period, and 12 months after initiating the hybrid intermittent regimen (1 mg 5d/7 with on-demand 2 mg dosing)

All 13 patients in Group A initially received sirolimus at 2 mg/day, administered five days per week with two non-consecutive rest days (Group A1). After six months, this intermittent regimen maintained pain control comparable to prior continuous sirolimus. Median pain intensity (VAS) decreased from 6 (range: 4–7) at baseline to 1 (range: 0–3) during continuous therapy, and remained stable at 1 (range: 0–3) during intermittent therapy (*p* = 0.55; Fig. 2A). Similarly, the frequency of monthly pain crises decreased from a median of 8 episodes (range: 8–15) pre-treatment to 2 episodes (range: 0–4) on continuous sirolimus, and remained at 2 episodes (range: 0–4) during intermittent administration (*p* = 0.65; Fig. 2B).

Following this phase, all patients transitioned to a reduced dose of 1 mg/day, maintaining the five days-ON/two days-OFF schedule (Group A2). After two months, three patients (23%) reverted to the original 2 mg/day intermittent regimen due to worsening pain. These patients had cervicofacial, lower limb, or thoracic VMs with unknown genetic drivers.

The remaining 10 patients continued on the 1 mg intermittent regimen. At six months (12 months after initiating intermittent therapy), median VAS scores remained stable at 2 (range: 0–3), with no significant increase in pain (*p* = 0.09). Pain crisis frequency also remained unchanged at 2 episodes/month (range: 0–3; *p* = 0.5), confirming the durability of symptom control (Figs. 2A and B).

Pain intensity (A) and crisis frequency (B) in Group A before sirolimus, three months after the continuous treatment period, and under stepwise intermittent dosing (Group A1: 2 mg 5d/7; Group A2: 1 mg 5d/7). Symptom control was maintained with reduced dosing. *** *p* < 0.001, NS non statistically significant Pain intensity (C) and crisis frequency (D) in Group B before sirolimus, three months after the continuous treatment period, and 12 months after initiating the hybrid intermittent regimen (1 mg 5d/7 with on-demand 2 mg dosing).

### Group B: hybrid strategy associating intermittent low dose and on-demand sirolimus

Group B included six patients treated with a hybrid regimen combining intermittent low-dose sirolimus (1 mg/day, five days-ON/two days-OFF) and on-demand dosing (2 mg) taken either before a known trigger or at the onset of a pain crisis. On-demand dosing was limited to a maximum of one additional dose per week. One patient affected by a VM with dilated veins of the lower limb with a *PIK3CA* pathogenic variant discontinued the hybrid regimen due to suboptimal symptom control and resumed continuous sirolimus at 2 mg/day. The remaining five patients continued the hybrid regimen and were evaluated at 12 months. Pain intensity significantly decreased from a pre-treatment median VAS of 5 (range: 4–6) to 2 (range: 0–3) during both continuous (*p* < 0.01) and hybrid (*p* < 0.01) sirolimus therapy (Fig. 2C). The number of monthly pain crises was also significantly reduced, from a median of 5 episodes (range: 3–5) before sirolimus to 1 episode (range: 0–2) on continuous treatment, and remained at 1 episode (range: 1–2) with the hybrid regimen (*p* < 0.01 for both). There was no statistically significant difference between continuous and hybrid administration for pain intensity (*p* = 0.55) or pain crisis frequency (*p* = 0.72; Figs. 2C and D).

### Group C1 (sirolimus on-demand in pain prevention)

Five patients with episodic pain and identifiable triggers, such as physical activity or anticipated exertion, followed an on-demand sirolimus regimen. They typically took 2 mg of sirolimus once or twice around trigger exposure, resulting in a dosing frequency of one to three times per week.

**Fig. 3:**
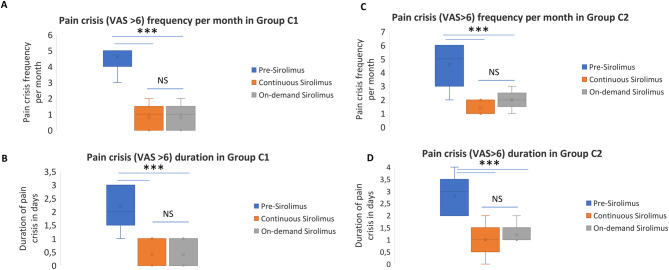
Evolution of symptoms on personalized sirolimus in group C. Frequency (**a**) and duration of pain crises (**b**), defined as pain intensity score superior to 6/10) in group C1 (on-demand before trigger) before sirolimus, three months after continuous treatment initiation, and 12 months after initiating the on-demand prophylactic sirolimus based on identified triggers. Intermittent prophylaxis maintained efficacy comparable to continuous dosing. Frequency (**c**) and duration of pain crises (**d**) in group C2 (on-demand at the beginning of the crisis) before sirolimus, three months after continuous treatment initiation, and 12 months after initiating the on-demand sirolimus at crisis onset. *** *p* < 0.001, ns non statistically significant

At 12-month follow-up, on-demand sirolimus was as effective as continuous therapy in reducing both the frequency and duration of pain crises. The median number of monthly crises decreased from 5 (range: 3–5) before treatment to 1 (range: 0–2) with on-demand therapy (*p* < 0.01), comparable to the effect seen with continuous sirolimus (*p* < 0.01). The median duration of pain crises also fell from 2 days (range: 1–5) to 0.5 days (range: 0–1) with both strategies (*p* < 0.01). There were no statistically significant differences between the two regimens for either outcome (Fig. 3A and B).

### Group C2 (sirolimus on-demand in stopping pain crisis)

Six patients with episodic pain—some without clear triggers—used sirolimus reactively at the onset of a pain crisis. Dosing frequency varied from once to twice weekly, typically initiated when VAS scores reached 4–5. One patient with a lower-limb LM resumed continuous sirolimus due to insufficient symptom control. Among the remaining five, median monthly crises decreased from 5 (range: 2–6) at baseline to 1 (range: 1–2) with continuous therapy (*p* < 0.01) and to 2 (range: 1–3) with on-demand therapy (*p* < 0.01). Although crisis frequency was numerically higher with the on-demand regimen, this difference was not statistically significant (*p* = 0.8). Additionally, both approaches shortened the duration of pain crises to a median of one day (*p* < 0.01), with no statistically significant difference between continuous and on-demand administration (*p* = 0.65) (Figs. 3C and D).

### Toxicity observed in the personalized regimen

Adverse event (AE) rates were substantially lower with intermittent and on-demand sirolimus regimens compared to prior continuous administration (Table 2).

**Table 2:** Profile of adverse events across the different regimens, according to CTCAE v5.0

	Group A (n = 13)						Group B (n = 6)				Group C (n = 11)			
	Continuous		Intermittent (A1)		Intermittent (A2)		Continuous		Intermittent		Continuous		Intermittent (C1 + C2)	
	**Grade 1/2**	**Grade 3/4**	**Grade 1/2**	**Grade 3/4**	**Grade 1/2**	**Grade 3/4**	**Grade 1/2**	**Grade 3/4**	**Grade 1/2**	**Grade 3/4**	**Grade 1/2**	**Grade 3/4**	**Grade 1/2**	**Grade 3/4**
All	10 (77)	1 (8)	4 (31)	0 (0)	1 (8)	0 (0)	5 (83)	0 (0)	2 (33)	0 (0)	8 (73)	0 (0)	1 (9)	0 (0)
Asthenia	8 (62)	0 (0)	3 (23)	0 (0)	1 (8)	0 (0)	4 (67)	0 (0)	2 (33)	0 (0)	7 (63)	0 (0)	1 (9)	0 (0)
Mucositis	6 (47)	1 (8)	3 (23)	0 (0)	0 (0)	0 (0)	3 (50)	0 (0)	1 (16)	0 (0)	4 (36)	0 (0)	0 (0)	0 (0)
Diarrhea	2 (15)	0 (0)	1 (8)	0 (0)	0 (0)	0 (0)	2 (33)	0 (0)	1 (16)	0 (0)	3 (27)	0 (0)	1 (0)	0 (0)
Skin rash	2 (15)	0 (0)	1 (8)	0 (0)	0 (0)	0 (0)	2 (33)	0 (0)	1 (16)	0 (0)	2 (18)	0 (0)	0 (0)	0 (0)
Headache	2 (15)	0 (0)	0 (0)	0 (0)	0 (0)	0 (0)	1 (16)	0 (0)	0 (0)	0 (0)	0 (0)	0 (0)	0 (0)	0 (0)

Number of patients experiencing adverse events, assuming that one patient may experience many adverse events

In Group A, 11 patients (85%) had experienced AEs during continuous sirolimus (10 with grade 1–2, and one with grade 3 mucositis). Under intermittent treatment, AE rates declined to 31% in Group A1 (all grade 1–2) and further to 8% in Group A2.

In Group B, continuous sirolimus was previously associated with an 83% AE rate (five patients, all grade 1–2). With the hybrid regimen, only 33% (two patients) experienced grade 1–2 AEs.

In Group C (C1 + C2), AE rates during continuous sirolimus were 73% (eight patients, all grade 1–2), whereas only one patient (9%) reported a grade 1 AE while on the on-demand regimen.

## Discussion

Long-term toxicity is a major concern in the management of slow-flow vascular malformations treated with sirolimus, particularly given the chronic nature of the disease and the frequent need for extended or repeated therapy. The VASE phase III trial, which mandated discontinuation of sirolimus after two years, provided a unique opportunity to reassess treatment needs and tailor re-treatment strategies based on individual symptom patterns and drug sensitivity.

This retrospective analysis demonstrates that intermittent, hybrid, and on-demand sirolimus regimens can effectively control symptoms—particularly pain—while significantly reducing toxicity in patients who previously benefited from continuous sirolimus. Notably, symptom control was maintained in the majority of patients across all adapted regimens, with no statistically significant loss of efficacy compared to continuous therapy.

In group A, intermittent regimen of 2 mg daily, five days-ON/two days-OFF provided pain management compared to continuous administration. Furthermore, nearly 80% of these patients were able to reduce their dose to 1 mg daily within this intermittent regimen, five days on 2 days off schedule, supporting the feasibility of dose de-escalation once symptoms are stabilized, offering a strategy to minimize drug exposure while maintaining benefit. In group C, the on-demand strategy, where sirolimus was taken either preventively (C1) or reactively (C2), outcomes were favorable, although a trend toward higher pain crisis frequency was noted in Group C2. Nevertheless, the benefit of this on-demand sirolimus intake remained significant compared to the pre-sirolimus period. The dosing schedule makes it challenging to determine whether the regular (though infrequent) use of sirolimus in these patients blurs the boundary between “on-demand” and “low-dose intermittent” therapy. The hybrid strategy (Group B) combining a low-dose baseline with occasional on-demand dosing appeared particularly promising. It maintained efficacy while reducing total drug exposure, and may represent a middle ground between strict on-demand use and more intensive continuous or intermittent regimens. Whether the pathogenic variant type and mutated gene (*TIE2* vs *PIK3CA*) influences the decision to adopt a specific intermittent regimen remains unknown.

The AE rate observed with continuous sirolimus administration was consistent with that reported in the VASE trial, confirming the reproducibility of its safety profile. Notably, all intermittent dosing regimens were associated with a substantial reduction in toxicity. This finding is particularly relevant in the context of long-term or chronic drug exposure, where cumulative adverse effects can significantly impact quality of life and adherence.

**Fig. 4:**
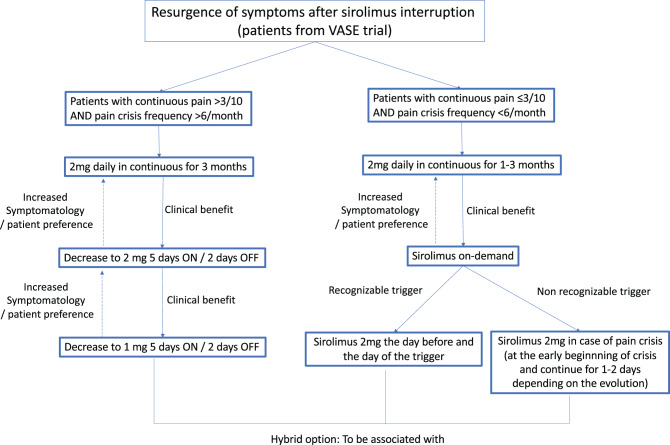
Proposed algorithm for personalized sirolimus dosing after treatment reintroduction. Patients are stratified based on pain characteristics into continuous, intermittent (5 days ON / 2 days off), or on-demand regimens, with flexibility to escalate or de-escalate dosing according to clinical response

Based on these findings, we propose a strategy for personalized sirolimus therapy (Fig. 4). The primary challenge is identifying which patients are more likely to benefit from a specific treatment regimen. On the other hand, as patients continue to respond to treatment, the dose can always be decreased whenever symptoms recur.

This study has several limitations. First, the sample size is small, primarily because only a limited percentage of patients experienced symptom recurrence after two years of sirolimus treatment. Second, patient selection was restricted to those capable of accurately describing their pain profile and adjusting their sirolimus intake accordingly. The applicability of this strategy in pediatric population remains uncertain. Additionally, our study focused on patients who had already demonstrated a positive response to sirolimus, as they had completed the two-year treatment within the VASE trial. This preselection inherently reflects a baseline sensitivity to sirolimus, which may not apply to all patients. Conversely, assessing intermittent or on-demand treatment strategies in sirolimus-naïve patients posed a greater challenge, as determining sirolimus sensitivity during the initial two-year treatment period was crucial for identifying the most appropriate regimen.

Future studies should aim to refine these strategies by identifying biomarkers that predict patient responses to specific regimens, ensuring a personalized approach to sirolimus therapy. Additionally, research should assess the long-term outcomes of intermittent sirolimus administration, including its impact on disease progression, quality of life, and cumulative toxicity reduction.

## Conclusion

In conclusion, this retrospective study suggests that personalized intermittent, hybrid, and on-demand sirolimus regimens may effectively control symptoms while reducing toxicity in patients with slow-flow vascular malformations who relapse after standard therapy. These findings support the feasibility of individualized, long-term management strategies and warrant prospective validation in larger and more diverse populations.

## Data Availability

The datasets used and/or analysed during the current study are available from the corresponding author on reasonable request.
